# Induction of Premature Labor

**Published:** 1862-07

**Authors:** C. D. Meigs

**Affiliations:** Emeritus Professor of Midwifery in Jefferson Medical College


*Induction of Premature Labor.* *By* C. D. MEIGS, M. D., *Emeritus Professor of Midwifery in Jefferson Medical College. In a letter to the Editor of the American Journal of the Medical Sciences.*

Dear Sir:—I am not rarely consulted by medical gentlemen at a distance on the subject of premature labor and on forced abortion supposed to be rendered necessary by an ascertained deformity of the pelvis. Having had two such letters to answer this week, I considered that it might comport with the purposes of your excellent journal to say in it what appears to me to be the most advisable method in such cases. If you should publish this note it might not only prove acceptable to some of your readers, but it would save my time, in the way of answering enquiries.

Dr. Karl Braun, of Vienna, proposed, a few years ago, to open the os uteri by means of what he called a *Colpeurynter*, which is a small, delicate bag or bladder composed of vulcanized rubber, to which is attached a syringe and a stop. This bladder, not bigger than a walnut, if introduced within the vagina, may be distended by means of the syringe with tepid water or air. It may be inflated or distended so as to become equal in size to a small fœtal head, and if the distension be gradually and very slowly effected it will not necessarily produce any distressing or painful sensation.

Now, as the uterine extremity of the vagina arises from the outer circumference of the cervix uteri, it is clear that such a distension as above supposed could not fail after due time to draw the walls of the cervical canal outwards, and so diminish the antagonism of the cervix and os uteri to that of the fundus and corpus uteri. This very condition of lessened resistance is all that is requisite in the case, because, as soon as the power of the fundus and body becomes preponderant, it will begin to force the ovum downwards, and at length, after dilating the cervix and os, expel it. I apprehend the method to be in all cases infallible—the condition being that the colpeurysis shall be properly effected.

To bring on a forced abortion in labor by the use of the stilette, or by injecting different medicinal articles or gases into the gravid womb, appears to me to be an encroachment, by medical people, upon those rules of art within whose sacred pale we may be held blameless, whereas, we are not without blame when we transcend those barriers that are so clearly defined in our collegiate diplomas. Witness the instant death that recently occurred at Edinburgh, from injecting carbonic acid into the uterus, as related in your last number! But to gently remove the retaining power of the os and cervix by a moderate and reasonable colpeurysis, is really to do no violence to any tissue, the operation being merely to suspend an antagonistic force, thus rendering the method of colpeurysis the simplest and safest possible, and certainly unfailing in success.

Inasmuch as operations performed for the induction of premature labor, and of abortion, are not without risk to the patient; and as it is well known that there are wretches who are so abandoned as to desecrate the profession of medicine, and spurn their own honor in the low calling *teneros avellere fœtus,* I conclude that no regular and reputable member of the body will ever consent to institute such operations as this I propose, without the consent of a proper medical consultation; this is a matter of such great import to us all, that I consider it to be incumbent on the American Medical Association to take such order upon it as their wisdom may suggest. To lose a patient under any circumstances, is a source of grief to the practitioner; but to lose one subjected, without consultation, to the operation in question, can only be fitly denoted as a disaster. Our diplomas endow us with authority to practice within the rules of an art—and it ought to be enacted as a rule of practice not to do these things without consent of a consultation. Such a rule would probably restrain much looseness of conduct on the part of some of the brethren; and might go even so far as to improve their morals, as is the admitted tendency of all salutary laws. I trust that you, Mr. Editor, who are a leader in things practical and æsthetical in our calling, will readily agree with me that it is neither safe nor decent for any single physician to order and perform one of these operations. Such sentiments, if known to be held by your great and powerful press, would do more good, perhaps, in the way of purging our brotherhood from these defilements, than half a dozen convictions and sentencings by the law courts.

Though I have already drawn out this note to a greater length than I designed, I ought not to omit the injunction of deferring the operation until after the period during which the ovum is covered up completely within its capsule called the decidua. Now, it is known that the whole ovum escapes from its deciduous investment or capsule at the one hundred and fifth day, or thereabout. If one would force on an abortion before that date, the deciduous capsule, which consists of the tubular glandular mucus membrane of the womb, must inevitably be ruptured or wounded, which is equal to a wound of the womb itself; but, after three and a half months the ovum, which is at that period wholly escaped from the decidua capsule, may reasonably be expected by means of the colpeurysis to be expelled whole and unbroken, in which case the risk to the woman is much diminished, not only because the entire product of the conception is thus eliminated, but also because no heedless and hazardous violence is done to the membrana decidua.

